# TLR-6 SNP P249S is associated with healthy aging in nonsmoking Eastern European Caucasians - A cohort study

**DOI:** 10.1186/s12979-016-0062-3

**Published:** 2016-03-17

**Authors:** Lutz Hamann, Jasmin Bustami, Leonid Iakoubov, Malgorzata Szwed, Malgorzata Mossakowska, Ralf R. Schumann, Monika Puzianowska-Kuznicka

**Affiliations:** Institute for Microbiology and Hygiene, Charité University Medical Center Berlin, Rahel-Hirsch-Weg 3, 10117 Berlin, Germany; Cellecta Inc, Mountain View, CA USA; Department of Human Epigenetics, Mossakowski Medical Research Centre, Polish Academy of Sciences, Warsaw, Poland; Polsenior Project, International Institute of Molecular and Cell Biology, Warsaw, Poland; Department of Geriatrics and Gerontology, Medical Centre of Postgraduate Education, Warsaw, Poland

**Keywords:** Healthy aging, *Inflamm-aging*, Toll-like receptor 6, Polymorphism

## Abstract

**Background:**

To investigate mechanisms that determine healthy aging is of major interest in the modern world marked by longer life expectancies. In addition to lifestyle and environmental factors genetic factors also play an important role in aging phenotypes. The aged immune system is characterized by a chronic micro-inflammation, known as *inflamm-aging*, that is suspected to trigger the onset of age-related diseases such as cardiovascular disease, Alzheimer’s disease, cancer, and Diabetes Mellitus Type 2 (DMT2). We have recently shown that a Toll-like receptor 6 variant (P249S) is associated with susceptibility to cardiovascular disease and speculated that this variant may also be associated with healthy aging in general by decreasing the process of *inflamm-aging*.

**Results:**

Analyzing the PolSenior cohort we show here that nonsmoking S allele carriers are significantly protected from age-related diseases (*P* = 0.008, OR: 0.654). This association depends not only on the association with cardiovascular diseases (*P* = 0.018, OR: 0.483) for homozygous S allele carriers, but is also driven by a protection from Diabetes Mellitus type 2 (*P* = 0.010, OR: 0.486) for S allele carriers. In addition we detect a trend but no significant association of this allele with inflamm-aging in terms of baseline IL-6 levels.

**Conclusion:**

We confirm our previous finding of the TLR-6 249S variant to be protective regarding cardiovascular diseases. Furthermore, we present first evidence of TLR-6 249S being involved in DMT2 susceptibility and may be in general associated with healthy aging possibly by reducing the process of inflamm-aging.

## Background

The process of successful aging and longevity is complex and far from being understood. Lifestyle, environment, and a limited aging-related impairment of the immune system (termed *immunosenescence*) are among the most important factors contributing to the healthy aging phenotype. The aged adaptive immune system is characterized by a decreased effectiveness due to decreased T- and B-cell responses and accumulation of functionally impaired memory lymphocytes [1]. Furthermore, the aged adaptive immune system is characterized by a dysregulation of T-cell subtypes, with a relative increase of Th2 lymphocytes resulting in increased levels of Th2 cytokines [2]. The aged innate immune system is characterized by reduced functions of neutrophils, macrophages, NK cells, and dendritic cells [3]. In addition, an important feature of the aged immune system is a chronic low grade inflammatory state, also known as micro-inflammation or inflamm-aging, which has been suspected to trigger aging-associated diseases such as cardiovascular disease (CVD), diabetes mellitus type 2 (DMT 2), metabolic syndrome, and neurodegeneration [4]. The mechanisms inducing the process of inflamm-aging are multifactorial and not completely understood. One reason may be the dysregulation of T lymphocyte subtypes. An enhanced translocation of gut bacteria as well as an enhanced DNA-damage in the elderly, which both trigger the innate pro-inflammatory immune response, are also discussed [5–7]. Furthermore, epigenetic changes in innate immune cells are discussed to be involved in inflamm-aging [8].

However, numerous genetic factors have also been proposed to influence aging. Several studies show that healthy aging and particularly longevity are highly heritable. For example, siblings of centenarians also have an enhanced probability for a longer healthy life [9, 10]. Genetic variations associated with the onset of age-related diseases such as atherosclerosis resulting in CVD, DMT 2, Alzheimer’s Disease, and cancer may play a pivotal role in determining successful aging [11]. Among aging-associated diseases, CVD plays a crucial role regarding mortality in the middle-aged population of the Western world [12, 13]. Recently, we have shown that the Toll-like receptor (TLR)-6 SNP P249S is associated with the risk for CVD with the homozygous S/S variant protecting from CVD. We also speculated whether this SNP may influence successful aging in general [14].

It has been shown that aging is associated with the dysregulation of expression and signal transduction of a variety of innate immune receptors, among them TLRs [15]. TLRs are important receptors of the innate immune system recognizing “pathogen associated molecular patterns (PAMPs)” followed by the activation of the innate immune response [16]. Although the process of inflammation is fundamental for survival in terms of combating infections or coping with damaging agents, it is also an important cause of many aging-associated diseases such as atherosclerosis, DMT 2, cognitive decline, and cancer, since these diseases are all associated with chronic inflammation [17]. The inflammatory response is driven by a variety of signaling pathways, however, the transcription factor NFκB has been considered to be the master regulator of inflammation [18], and is also a central part of TLR signaling [16].

Since inflamm-aging seems to be one of the reasons for the development of aging-related diseases, we postulated previously that variations within the TLR-system that decrease the inflammatory response may lead to a decreased process of inflamm-aging and may be, therefore, beneficial in terms of healthy aging. First evidence for this hypothesis was given by the finding that such variations are overrepresented in healthy elderly subjects in comparison to healthy younger subjects [19]. Following this discovery, we here investigated a cohort of elderly Caucasians and show that in nonsmoking elderly subjects the TLR-6 SNP P249S is associated with healthy aging. Analysis of certain aging related diseases revealed a significant protective effect on CVD and DMT 2.

## Methods

### Study subjects

A sub-group of 1544 participants of the PolSenior program, the first ones for whom the complete medical records (including, among others, data on cardio-vascular and respiratory diseases, cancer, diabetes, stroke and cognitive impairment) and DNA samples were available at the beginning of current study, was analyzed. PolSenior was a multicenter, interdisciplinary project, designed to assess health and socio-economic status of the Polish Caucasians aged ≥65 years. Details of the PolSenior recruitment are described elsewhere [20]. Project participants completed a detailed questionnaire regarding their medical, social, and economic past and current health status, underwent an examination including elements of comprehensive geriatric assessment, and donated blood for biochemical and genetic analyses. Steady state IL-6 level were measured using ELISA method (R&D System, Minneapolis, MN, USA, sensitivity 0.04 pg/mL). Blood pressure was measured three times during the first and second visit using validated, automatic blood pressure measuring device (A&D UA 787, A&D Company Limited, Tokyo, Japan) in a seated position. The study subject was diagnosed as suffering from hypertension if his/her average blood pressure from these measurements was ≥ 140/90 mmHg, or the disease has been previously diagnosed and the patient was taking hypotensive drugs over the past two weeks. The study was approved by the Bioethics Commission of the Medical University of Silesia in Katowice. All participants gave a written, informed consent for participation in the study. All investigations were carried out in accordance with the ethical guidelines of the 1975 Declaration of Helsinki. Subjects with MMSE < 24, determined according to the Polish version of the Mini-Mental State Examination test, were classified as subjects with cognitive impairment. According to Akbaraly et al. we set IL-6 levels ≤1.0 ng/L as low, 1.1–2.0 ng/L as intermediate, and >2 ng/L as high [21]. Low IL-6 levels were used as reference in logistic regression analysis.

### Genotyping

Genomic DNA was prepared by standard procedures from whole blood. Genotyping for TLR-6 SNP P249S (rs5743810) was performed by melting curve analysis as described previously [14]. Melting curve analysis was carried out employing primers gaaagactctgaccaggcat (forward), ctagtttattcgctatccaagtg (reverse), and FRET-hybridization probes: accagaggtccaaccttactgaa-FL and LC-red 640-ttaccctcaaccacatagaaacgacttgga resulting in melting points of 61, and 52 °C for the wild-type and the mutated allele, respectively.

### Statistics

Binary logistic regression analyses, Mann–Whitney U-test, Kruskal-Wallis, and T-test have been performed employing the IBM SPSS Statistics software package (version 20.0, IBM, Munich, Germany).

## Results

### Association of common risk factors with age related diseases

First we analyzed whether common risk factors such as age, BMI, micro-inflammation (presented as IL-6 levels), smoking, and hypertension are associated with aging-related diseases in our cohort of elderly Caucasians by comparison of healthy subjects without any aging-related disease (*n* = 517), and subjects suffering from one or more aging-related diseases (*n* = 1027), e.g. CVD (*n* = 496), cancer (*n* = 93), lung disease (*n* = 254), DMT 2 (*n* = 122), and cognitive impairment (*n* = 406). Baseline characteristics of the cohort are shown in Table 1. As expected, increased age and BMI were strongly associated with the occurrence of aging-related diseases (*P* < 0.001 for both). Also inflamm-aging, determined by baseline IL-6 levels with levels ≤1 ng/L serving as reference and 1.1–2.0 ng/L as intermediate or >2 ng/L as high levels, could be shown to be a strong risk factor (*P* = 0.016 and *P* < 0.001, respectively). Hypertension, interestingly, failed to be a significant risk factor in our cohort. Surprisingly, current smoking revealed a significant “protective” effect (*P* = 0.003, OR: 0.586), whereas past smoking showed no association (Table 2). However, further analysis of the distribution of age and BMI in nonsmokers, past smokers, and current smokers revealed current smokers to be significantly younger and having a significantly lower BMI, which might account for the “protective” effect of current smoking (data not shown). Indeed, performing a multivariate analysis with inflamm-aging, age, BMI and smoking as risk factors revealed that micro-inflammation - presented as intermediate or high IL-6 levels -, age and BMI remained significant independent risk factors in our elderly cohort, with *P*-values of 0.034, 0.001, 0.001, and 0.008, respectively. Past smoking and current smoking in a multivariate analysis failed to be significantly associated with aging-related diseases (Table 3).

**Table 1:** Baseline characteristic of the study subjects

PolSenior group	
*N*	1544
Age range	61–92
Mean age (SD)	76.76 (6.44)
Males/females	811/733
Mean BMI (SD)	28.46 (4.87)
Smoking: never/past/current (%)	849 (55.0)/534 (34.6)/152 (9.8)
Hypertension yes/no (%)	1167/372 (75.6/24.1)
Baseline IL-6 (ng/L)	3.21 (2.98)
Disease-free	517 (33.5)
CVD (%)	496 (32.1)
Cancer (%)	93 (6.0)
Lung disease (%)	254 (16.5)
DMT 2 (%)	122 (7.9)
Cognitive impairment (%)	406 (26.3)
TLR-6 PP/PS/SS (%)	615 (39.8)/718 (46.5)/211 (13.7)

**Table 2:** Association of common risk factors with aging-related diseases in the PolSenior group. Univariate analysis of common risk factors and aging related diseases

Risk factor		*P*-value	Mean (SD)
Age		<0.001	75.7 (6.6)/77.3 (6.3)
BMI		<0.001	27.9(4.8)/28.6 (4.9)
		*P*-value	OR (95 % CI)
IL-6	Intermediate	0.016	1.578 (1.088–2.289)
	High	<0.001	2.108 (1.472–3.020)
Smoking	Past	0.619	1.061 (0.841–1.338)
	Current	0.003	0.586 (0.413–0.831)
Hypertension		0.175	1.184 (0.928–1.512)

Age was analyzed by T-test and BMI was analyzed by Mann–Whitney U test. Micro-inflammation (IL-6), smoking and hypertension were analyzed by logistic regression with IL-6 levels ≤1 ng/L as reference and 1.1–2.0 ng/L (intermediate) and >2 ng/L (high) as predictors, as well as no smoking or no hypertension as reference

**Table 3:** Association of common risk factors with aging-related diseases in the PolSenior group. Multivariate analysis of common risk factors and aging-related diseases

Risk factor		*P*-value	OR (95 % CI)
Age		0.001	1.033 (1.014–1.052)
BMI		0.008	1.033 (1.009–1.058)
IL-6	Intermediate	0.034	1.507 (1.031–2.202)
	High	0.001	1.863 (1.286–2.700)
Smoking	Past	0.310	1.134 (0.890–1.446)
	Current	0.052	0.683 (0.465–1.003)

Analysis was done by logistic regression with IL-6 levels ≤1 ng/L as reference and 1.1–2.0 ng/L (intermediate) and >2 ng/L (high) as predictors for inflamm-aging, and no smoking as reference

### The TLR-6 variant 249S/S is significantly associated with protection from aging-related diseases in non-smoking subjects

Having shown that common risk factors (with the exception of smoking) exhibited the expected association with aging-related diseases in our cohort, we next analyzed whether the TLR-6 P249S variation is also associated with such diseases. Analyzing the whole cohort by logistic regression, using the wild-type genotype as reference we failed to find any significant association. Correction for age, BMI and inflamm-aging was done by adding these factors as co-factors in the regression analysis (Table 4). Since in this population smokers are significantly younger and exhibit a significantly lower BMI the pattern of association of smoking with diseases could not be analyzed in detail, and in order to avoid further statistical problems, only non-smokers were selected for association studies of TLR-6 polymorphism with aging-related diseases.

**Table 4:** TLR-6 P249S is associated with healthy aging in non-smokers

Disease	Variant	*P*-value	OR (95 % CI)
All subjects			
Combined aging related diseases	P/P	-	-
	P/S	0.281	0.876 (0.689–1.114)
	S/S	0.273	0.825 (0.585–1.163)
	P/S + S/S	0.208	0.864 (0.689–1.085)
Non smokers			
Combined aging related diseases	P/P	-	-
	P/S	0.024	0.682 (0.489–0.951)
	S/S	0.018	0.566 (0.353–0.909)
	P/S + S/S	0.008	0.654 (0.477–0.897)

*P*-values and OR were determined by logistic regression using the wild type genotype as reference, and micro-inflammation (IL-6 level), age and BMI as co-factors. Subjects with a white blood cell count above 10.000/μl were excluded

Comparing non-smoking healthy subjects (*n* = 278) with non-smoking diseased subjects (*n* = 571) we found a significant protective association of the TLR-6 P/S and TLR-6 S/S variants with aging-related diseases in general, *P* = 0.024, OR: 0.682 (95 % CI: 0.489–0.951) and *P* = 0.018, OR: 0.566 (95 % CI: 0.353–0.909), respectively. The protective effect for mutated allele carriers was still in effect when heterozygous and homozygous genotypes were combined, *P* = 0.008, OR: 0.654 (95 % CI: 0.477–0.897) (Table 4), which is compatible with the dominant model. No significant effects were found in formerly or currently smoking subjects, which, as stated above, may be due to the different age-distribution of these groups (data not shown). As shown in Table 5, a subgroup analysis of individual diseases revealed a significant protective effect of the TLR-6 S/S variant regarding CVD (*n* = 255), *P* = 0.018, OR: 0.483 (95 % CI: 0.265–0.882). Correction for age, BMI and inflamm-aging was done by adding these factors as co-factors in the regression analysis. Interestingly, the heterozygous P/S variant as well as the combined heterozygous and homozygous genotypes exhibited a significant protection regarding DMT 2 (*n* = 79), *P* = 0.007 OR: 0.443 (95 % CI: 0.244–0.804) and *P* = 0.010 OR: 0.486 (95 % CI: 0.281–0.839), respectively. For cancer (*n* = 42), lung disease (*n* = 116), and cognitive impairment (*n* = 250), no significant associations were found. Analysis of combined aging-related diseases with exclusion of CVD (*n* = 309) revealed significant protection for the TLR-6 P/S variant, *P* = 0.016 OR. 0.628 (95 % CI: 0.431–0.915) and the combined heterozygous and homozygous genotypes, *P* = 0.012, OR: 0.635 (95 % CI: 0.445–0.906) indicating a protective effect independent of the protection against CVD.

**Table 5:** TLR-6 P249S is associated with CVD and DMT 2 in non-smokers

Disease	Variant	*P*-value	OR (95 % CI)
All subjects			
CVD	P/P	-	-
	P/S	0.188	0.770 (0.522–1.136)
	S/S	0.018	0.483 (0.265–0.882)
	P/S + S/S	0.061	0.702 (0.484–1.017)
Cancer	P/P	-	-
	P/S	0.416	0.745 (0.367–1.513)
	S/S	0.353	0.602 (0.207–1.756)
	P/S + S/S	0.318	0.710 (0.363–1.390)
Lung disease	P/P	-	-
	P/S	0.216	0.732 (0.446–1.201)
	S/S	0.509	0.793 (0.399–1.577)
	P/S + S/S	0.219	0.746 (0.467–1.191)
DMT 2	P/P	-	-
	P/S	0.007	0.443 (0.244–0.804)
	S/S	0.230	0.617 (0.281–1.356)
	P/S + S/S	0.010	0.486 (0.281–0.839)
Cognitive impairment	P/P	-	-
	P/S	0.284	0.794 (0.522–1.210)
	S/S	0.345	0.751 (0.415–1.360)
	P/S + S/S	0.232	0.784 (0.526–1.1168)
Combined aging related diseases, CVD excluded	P/P	-	-
	P/S	0.016	0.628 (0.431–0.915)
	S/S	0.116	0.656 (0.388–1.109)
	P/S + S/S	0.012	0.635 (0.445–0.906)

*P*-values and OR were determined by logistic regression using the wild type genotype as reference, and micro-inflammation (IL-6 level), age and BMI as co-factors. Subjects with a white blood cell count above 10.000/μl were excluded

### No association of TLR-6 6 P249S variation with baseline IL-6 levels

Since we have speculated previously that a beneficial effect of TLR SNPs may rely on a decreased process of inflamm-aging due to a reduced sensitivity of the innate immune system, we analyzed whether the TLR-6 P249S variant was associated with baseline levels of IL-6. To exclude current infections that could affect the level of IL-6, we excluded subjects with a white blood cell count above 10.000/μl. As shown in Table 6, baseline IL-6 levels are significantly higher in subjects suffering from one or more aging-related diseases in comparison to healthy subjects: 3.33 ng/L vs. 2.75 ng/L, *P* >0.000. Although there is a trend for decreased IL-6 baseline levels in S allele carriers, P/P: 3.23 ng/L, P/S: 3.08 ng/L and S/S: 3.05 ng/L, this association is not significant, *P =* 0.850.

**Table 6:** TLR-6 P249S is not associated with baseline levels of IL-6

	IL-6 (ng/L, mean, SD)	*P*-value
Disease free	2.75 (2.60)	<0.001
Aging related diseases	3.33 (3.01)	
TLR-6 genotype		
P/P	3.23 (3.05)	0.850
P/S	3.08 (2.73)	
S/S	3.05 (2.93)	

*P*-values were determined by Mann–Whitney U test or Kruskal-Wallis test, subjects with white blood cells above 10.000/μl were excluded

## Discussion

We have recently shown that TLR-6 P249S is associated with coronary artery disease, one of the most important aging-related diseases, and have speculated that this genetic variation may be also associated with healthy aging in general by decreasing the process of inflamm-aging due to decreased sensitivity of the innate immune response [14, 19]. Healthy, or successful aging is affected by a variety of factors including environmental factors, life style, micro-inflammation, and genetic factors that determine the occurrence, onset and course of aging-related diseases. Since most age-related diseases are associated with chronic inflammation [21], a sensitive immune system may be an example of antagonistic pleiotropy with beneficial effects at early age but adverse effects in later life since evolution optimizes for fitness, not for longevity [22]. By analyzing a middle-aged population-based cohort, exhibiting the typical distribution of aging-related diseases, we here confirmed our previous finding of TLR-6 P249S being associated with CVD and showed that this SNP is associated with healthy aging in non-smoking subjects of the PolSenior cohort. This positive association with healthy aging remains significant after exclusion of subjects suffering from CVD indicating a more general effect, not only restricted to CVD. Furthermore we present first evidence of this SNP to be also involved in DMT-2 and the process of inflamm-aging in non-smoking subjects.

We have currently no conclusive explanation why this effect could not be shown in smoking subjects. Although our study does not show smoking to be one of the strongest risk factors for aging-related diseases in individuals 65 years old and older, the effect of TLR-6 variants on healthy aging may nevertheless be masked by smoking. Such modifying effect of smoking has recently been reported for several genetic associations in age-related macular degeneration which are largely restricted to non-smokers [23]. One possible explanation could be that in our cohort smoking is associated with both, lower ager and lower BMI which in turn are associated with the absence of aging-related diseases. The association of TLR-6 P249S with DMT 2 has not been shown before. However, since the number of non-smoking subjects with DMT 2 was rather low in our cohort (*n* = 79), and TLR-6 has not been found by GWAs for DMT 2, these findings have to be confirmed in larger studies [24].

Analyzing the whole cohort for common risk factors by multivariate regression analysis showed, as expected, increased age, BMI, and inflamm-aging to be strongly associated with the occurrence of aging-related diseases. Since we have previously speculated that less functional TLR variants decreasing the sensitivity of the innate immune system may dampen the process of inflamm-aging, we analyzed the association of TLR-6 P249S with baseline IL-6 levels. Although the S allele at position 249 of TLR-6 has been shown to decrease TLR-6 signaling [25], we could not find a significant effect on inflamm-aging. However, we could show a trend towards decreased IL-6 levels in S allele carriers. The failure of finding significant changes may potentially be explained by the fact that other pro-inflammatory cytokines such as TNF-α and IL-1β, the serum-levels of which were not available to us, may be more involved in the process of inflamm-aging as compared to IL-6. Recent data show that inflamm-aging is not simply the chronic increase of pro-inflammatory markers such as IL-6 and CRP. Inflamm-aging seem to be much more complex and is characterized by the simultaneous change in both pro- and anti- inflammatory markers [26]. Furthermore, repeated measurements of IL-6 levels, which were not available to us in this cohort are known to be more accurate in determining inflamm-aging [2]. Finally, the SNPs investigated by us may lead to changes in cytokines in certain tissues, which cannot be monitored by measuring serum levels.

On the other hand, comparing healthy subjects with diseased subjects, a strong correlation of increased IL-6 baseline levels and aging-related diseases was shown. This may be evidence for the fact that inflamm-aging is driven mainly by a dysregulated T lymphocyte subpopulation [2] and chronic stimulation of the innate immune system via TLRs does not play the dominant role.

Since the limitation of our study is relatively small sample number, especially in disease subgroups, our results require further investigation by independent studies.

## Conclusion

In conclusion we confirm here our previous results of less functional TLR-6 variant 249S to protect from CVD. In addition we show here that this variant protects also from DMT-2 and is in general associated with healthy aging in the PolSenior cohort. Furthermore, we could show a trend for a decreased process of inflamm-aging by the TLR-6 249S allele as measured by IL-6 levels. However, the effect of TLR variants on the process of *inflamm-aging* needs to be confirmed by further studies.
